# Robust and Reproducible *Agrobacterium*-Mediated Transformation System of the C_4_ Genetic Model Species *Setaria viridis*

**DOI:** 10.3389/fpls.2020.00281

**Published:** 2020-03-13

**Authors:** Duc Quan Nguyen, Joyce Van Eck, Andrew L. Eamens, Christopher P. L. Grof

**Affiliations:** ^1^Centre for Plant Science, School of Environmental and Life Sciences, The University of Newcastle, Callaghan, NSW, Australia; ^2^Boyce Thompson Institute, Ithaca, NY, United States; ^3^Plant Breeding and Genetics Section, School of Integrative Plant Science, Cornell University, Ithaca, NY, United States

**Keywords:** C_4_ monocotyledonous grasses, *Setaria viridis*, *Agrobacterium tumefaciens*, *Agrobacterium-*mediated transformation, stable transformants, *in planta* transgene selection

## Abstract

*Setaria viridis* (green foxtail) has been identified as a potential experimental model system to genetically and molecularly characterise the C_4_ monocotyledonous grasses due to its small physical size, short generation time and prolific seed production, together with a sequenced and annotated genome. *Setaria viridis* is the wild ancestor of the cropping species, foxtail millet (*Setaria italica*), with both *Setaria* species sharing a close evolutionary relationship with the agronomically important species, maize, sorghum, and sugarcane, as well as the bioenergy feedstocks, switchgrass, and *Miscanthus*. However, an efficient and reproducible transformation protocol is required to further advance the use of *S. viridis* to study the molecular genetics of C_4_ monocotyledonous grasses. An efficient and reproducible protocol was established for *Agrobacterium tumefaciens*-mediated transformation of *S. viridis* (Accession A10) regenerable callus material derived from mature seeds, a protocol that returned an average transformation efficiency of 6.3%. The efficiency of this protocol was the result of the: (i) use of mature embryo derived callus material; (ii) age of the seed used to induce callus formation; (iii) composition of the callus induction media, including the addition of the ethylene inhibitor, silver nitrate; (iv) use of a co-cultivation approach, and; (v) concentration of the selective agent. Our protocol furthers the use of *S. viridis* as an experimental model system to study the molecular genetics of C_4_ monocotyledonous grasses for the potential future development of improved C_4_ cropping species.

## Introduction

The C_4_ monocotyledonous (monocot) grasses contribute a large proportion of global food production for both human and animal consumption, as well as biomass for biofuel production (Vogel, 2008; Kellogg, 2013; OECD-FAO, 2017). With the ever-increasing demand for the continued generation of a reliable source of food and fuel production, efforts to continue to advance C_4_ grass crop yield and to improve plant biomass quality are of critical importance. Therefore, an urgent requirement exists to develop an experimental model system to genetically characterise C_4_ photosynthesis as well as to further our current molecular understanding of the development of C_4_ monocot grass species.

*Setaria viridis* (green foxtail) has been identified as a promising experimental model for the genetic and molecular characterisation of the C_4_ monocot grasses. *Setaria viridis* (*S. viridis*) is the wild ancestor of the cropping species, *Setaria italica* (foxtail millet), with both *Setaria* species sharing a close phylogenetic relationship to many agronomically important C_4_ crops, including *Zea mays* (maize), *Sorghum bicolor* (sorghum) and *Saccharum officinarum* (sugarcane), and to the bioenergy feedstocks, *Panicum virgatum* (switchgrass) and *Miscanthus* × *giganteus* (Li and Brutnell, 2011; Brutnell et al., 2010; Sebastian et al., 2014). *S. viridis* also possesses additional attributes that have identified it as a promising experimental model system for the genetic characterisation of the C_4_ grasses, including its small physical size (10–15 cm in height), rapid life cycle (6–9 weeks from seed to seed), prolific seed production (∼13,000 seeds per plant) and simple growth requirements (Li and Brutnell, 2011). More importantly, *S. viridis* is supported with a sequenced and annotated genome (Li and Brutnell, 2011; Bennetzen et al., 2012; Sebastian et al., 2014), and a comprehensive transcriptome of an elongating internode (Martin et al., 2016) and an expanding leaf (Studer et al., 2016). The availability of these genetic tools for *S. viridis* has facilitated studies on the complex C_4_ gene networks, studies that resulted in the identification and characterisation of several gene families involved in cell wall biosynthesis, such as *Cellulose synthase* genes (CesA and Csl; Ermawar et al., 2015; Muthamilarasan et al., 2015), and monolignol synthesis genes (Ferreira et al., 2019). The potential use of *S. viridis* as an experimental model has led to the required development of an efficient and reproducible transformation protocol to enable the continued molecular investigation into the complex gene networks that control C_4_ photosynthesis and cell wall development.

Over the last three decades, several transformation protocols have been developed for the genetic manipulation of a range of plant species, including electroporation (Fromm et al., 1985; Saunders et al., 1995), particle bombardment (Klein et al., 1988; Yao et al., 2006; Liu et al., 2014), and more recently, the use of either *Agrobacterium tumefaciens* or *Agrobacterium rhizogenes* (Hohn et al., 1989; Wu et al., 2013; Liu et al., 2015). Furthermore, a range of explant material, including immature embryos, whole seeds, anthers, immature inflorescences and callus derived from these different explant materials, was identified in parallel as the suitable transformation material dependent on the transformation protocol being applied (Carvalho et al., 1997; Nishimura et al., 2006; Song et al., 2011; Wang et al., 2011). Via exploitation of the natural gene transfer capability of *A. tumefaciens* and *A. rhizogenes*, the *Agrobacterium*-mediated system offers several advantages over other transformation approaches, namely the routine obtainment of high transformation efficiencies, low transgene copy number integration events, clean and intact integration of relatively large segments of introduced DNA, and the stable inheritance and expression of introduced transgenes (Chan et al., 1993; Shrawat and Good, 2011; Krishnan et al., 2013).

Like other members of the Panicoideae subfamily of C_4_ monocot grasses, *S. viridis* is not a natural host of *Agrobacterium* (Azria and Bhalla, 2000; Shrawat and Lorz, 2006), therefore rendering the establishment of an efficient and reproducible transformation protocol for *S. viridis* highly challenging. In 2015, the Van Eck and Monilari research groups first reported the successful *Agrobacterium*-mediated transformation of *S. viridis* via the use of a tissue culture based mature seed derived callus approach (Martins et al., 2015a; Van Eck and Swartwood, 2015), or a non-tissue culture based floral dip method using immature inflorescences (Martins et al., 2015b).

Here we report on an improved protocol for the efficient and reproducible generation of stable *S. viridis* transformant lines via an *Agrobacterium*–mediated transformation approach. The improved efficiency of the protocol described here was the result of the; (1) use of embryo derived callus material that was generated from dehusked mature seed; (2) age of the seed used for callus induction; (3) density of seed per callus induction plate; (4) composition of the callus induction medium, including the addition of the ethylene inhibitor, silver nitrate; (5) use of a co-cultivation transformation approach, and; (6) concentration of the selective agent at the plant regeneration stage. Together, the optimisation of each of these factors allowed for a maximum transformation efficiency of 6.3% to be routinely achieved.

## Materials and Methods

### Plant Material

*Setaria viridis* (L.) Beauv. (Accession A10) was used for all experimental work performed in this study. The seeds of a wild-type *S. viridis* plant had been stored for at least 1 month before they were sown under a thin layer of a soil mixture composed of coarse sand, coco peat and perlite at a ratio of 2:1:1. Post planting of the wild-type seeds, the germinated wild-type *S. viridis* plants were cultivated under a controlled growth regime of 16 h of light (∼600 μmol.m^–2^.s^–1^), 8 h of darkness, and a day/night temperature of 28°C/20°C, until the plants completed their developmental cycle. Mature seeds were harvested, air-dried at 37°C for 7 days, and then stored at 4°C.

### Callus Induction

For callus induction, 1-year-old mature seeds were de-husked, and surface-sterilised with 10% (v/v) commercial bleach and 0.1% (v/v) Tween-20 for 5 min with gentle rotation. The seeds were next washed three times with sterilised distilled water via gentle hand inversion. Post washing, the seeds were air-dried on sterilised Whatman^®^ filter paper in a biosafety cabinet. In the biosafety cabinet, 18 dried seeds were transferred to each culture plate that contained callus induction medium (CIM) supplemented with 5.0 mg/L of silver nitrate (see Supplementary Appendix S1). Culture plates were maintained in the dark for 4 weeks at 24°C and 70% relative humidity. At the end of the initial 4-week induction period, good quality regenerable callus, which was yellow in colour and consisted of a compact mass of cells, was subsequently subcultured onto fresh CIM medium every 15 days. The subculturing step was only performed a maximum of four times to ensure that genomic alterations were not being introduced into the *S. viridis* callus material (Plaza-Wüthrich and Tadele, 2012) prior to its use as explant material for *Agrobacterium*-mediated transformation.

### Plasmid and Bacterial Strain

A Gateway^TM^ compatible pANIC12A vector (Mann et al., 2012a) was used for all experimental work performed in this study (see Supplementary Appendix S2). The pANIC12A plasmid (obtained from the Arabidopsis Biological Resource Center, Stock number: CD3-1720) was transferred to *Agrobacterium tumefaciens* (*Agrobacterium*) strain AGL1 via electroporation, and the strain harbouring the plasmid was preserved at −80°C.

### Preparation of the *Agrobacterium* Culture for Co-cultivation

An aliquot of *Agrobacterium* harbouring the pANIC12A plasmid was used to inoculate 5.0 mL of YEP liquid medium (see Supplementary Appendix S1). This primary culture was incubated at 28°C on a rotating platform for 36 h. Five hundred microlitres of the primary culture was used to inoculate 100 mL of fresh YEP liquid medium, and this working culture preparation was incubated at 28°C on a rotating platform until the *Agrobacterium* reached the exponential growth phase [0.6 optical density at wavelength 600 nanometres (OD_600__nm_)]. The bacterial cells were pelleted by centrifugation for 10 min at 8000 × *g* at 20°C. The pelleted cells were resuspended in 100 mL of liquid co-cultivation medium (CIMC) (see Supplementary Appendix S1), and immediately prior to co-cultivation with the callus material, 100 μL of 200 μM acetosyringone and 1.0 mL of Synperonic^®^ (10% w/v) were added to the liquid CIMC medium to enhance the efficiency of transformation (Jambagi et al., 2010; Karthikeyan et al., 2011).

### *Agrobacterium*-Mediated Transformation of *Setaria viridis* Callus

Healthy pieces of callus were transferred to a sterilised conical flask that contained 100 mL of the *Agrobacterium* suspension as described above. The calli were submerged in the *Agrobacterium* suspension for 5 min at room temperature, and the flask was gently and briefly shaken by hand each minute of the 5-min incubation period to ensure that all callus pieces were evenly distributed in the suspension. The calli were next transferred to sterilised filter paper and allowed to dry at room temperature for 5 min before being transferred to culture plates containing solid CIMC medium. The surface of the solid CIMC medium was covered with a single layer of Whatman^®^ filter paper to prevent direct contact with the transfected callus. All plates were tightly sealed with 3M^TM^ porous tape and maintained in the dark at 24°C for 3 days. After 3 days of co-cultivation, calli underwent two, 10-day rounds of screening via their transferral to solid selective medium (CIMS) (see Supplementary Appendix S1). At the completion of the second round of selection, only hygromycin-resistant calli were transferred to fresh culture plates that contained selective plant regeneration medium (PRMS) (see Supplementary Appendix S1). The calli were maintained at 24°C in the light (150–200 μmol.m^–2^.s^–1^) for 20 to 30 days, with subculturing to fresh medium every 10 days. Once the shoot material which had emerged from the callus reached 1.0 to 3.0 cm in length, the entire shoot and callus structure was transferred to an individual Magenta^TM^ vessel that contained selective root induction medium (RMS) (see Supplementary Appendix S1). The shooting calli were maintained on solid RMS medium for 15–20 days at 24°C, under a 16/8-h light/dark photoperiod and at 70% humidity. Each plantlet that formed from each suspected individual transformation event was transferred to a small labelled pot (5.0 cm in diameter) that contained a 2:1:1 mixture of coarse sand, coco peat, and perlite. Transferred plantlets were cultivated under transparent plastic covers to maintain high humidity for the first 5–7 days after soil transfer, for acclimation. Acclimated plantlets were then transferred to larger sized labelled pots (20 cm in diameter) that contained the same soil mixture and were cultivated under standard glasshouse conditions (16/8-h light/dark photoperiod and a 28°C/20°C day/night temperature) for seed production.

### Visualisation of RFP

RED FLOURESCENT PROTEIN (RFP) expression was visualised using a Leica Fluo III fluorescence microscope (Leica, Wetzlar, Germany) and an Olympus IX81 confocal microscope (Olympus, Tokyo, Japan) equipped with an RFP filter set (excitation wavelength 580 nm and emission wavelength 630 nm). For visualisation of RFP fluorescence in callus material 5-days post co-cultivation with *Agrobacterium*, callus remained in sealed culture plates, however, RFP expression was visualised in internode and root samples via the mounting of these plant materials on glass slides. Wild-type material that had not been co-cultivated with *Agrobacterium* harbouring the pANIC12A vector, was used as the negative control for RFP investigation. All images were collected under bright field illumination, and via the use of the same RFP filter set and microscopy settings.

### Isolation of Genomic DNA

Genomic DNA was extracted from *S. viridis* tissues using Cetyltrimethyl ammonium bromide (CTAB) extraction buffer. This protocol was based on the methodology reported by Doyle and Doyle (1990) with minor modifications for optimisation. The concentration of all DNA samples was measured using a Nanodrop^®^ 1000 spectrophotometer (Thermo Fisher Scientific, Massachusetts, United States), and all genomic DNA extracts were stored at −20°C until required for use in subsequent experimental analyses.

### Southern Blot Hybridisation Analysis

Southern blot hybridisation analysis was conducted on successive transformant generations to confirm that the pANIC12A derived T-DNA had stably integrated into the genome of each *S. viridis* transformant line, and post transgene integration, stable inheritance of the introduced transgene. Two plants from each independent transformant line were used for Southern blotting. Genomic DNA samples were digested with the restriction endonuclease *Pst*I (New England Biolabs, Queensland, Australia) for 8 h at 37°C. The *Pst*I-digested genomic DNA was next fractioned on a 1.0% (w/v) TBE agarose gel (see Supplementary Appendix S3), and then capillary blotted onto a positively charged Zeta-Probe nylon membrane (Bio-Rad, CA, United States) according to the manufacturer’s instructions. The DNA was pre-hybridised in modified Church and Gilbert solution (see Supplementary Appendix S3) at 55°C for 1 h. A column purified (Qiagen, Victoria, Australia) PCR product of the hygromycin resistance gene (*HPTII*) harboured by the T-DNA of the pANIC12A plant expression vector (the *HPTII* amplicon was amplified from a pANIC12A plasmid template using the forward primer, 5′-GTGCTTGACATTGGGGAGTT-3′; and reverse primer, 5′-CGTCTGCTGCTCCATACAAG-3′) was used as the template to produce a radiolabelled probe. The *HPTII*-specific radiolabelled probe was prepared via the addition of 0.75 MBq [α-^32^P]-dCTP (PerkinElmer, Victoria, Australia) to the probe mixture [200 ng DNA template; 50 ng random hexamer primer; 1 X NEB Buffer 2.1; 0.05 mM dATP/dTTP/dGTP mixture; 5.0 units of Klenow Fragment (New England Biolabs, Queensland, Australia)], and incubation at room temperature for 1 h. The probe was denatured by boiling at 95°C for 5 min, before quickly chilling on ice for 5 min. The denatured *HPTII*-specific radiolabelled probe was added to the pre-hybridised membrane, and the membrane was incubated overnight at 55°C in a HybriLinker hybridisation oven (UVP, California, United States) with constant rotation. The hybridised membrane was then washed 3–6 times with washing buffer of three different stringencies for 5 min per wash (see Supplementary Appendix S3). The probed membrane was then exposed on a Phosphor Screen overnight, and the resulting hybridisation signals were visualised using a Typhoon Scanner (GE Healthcare Life Sciences, Illinois, United States).

### Statistical Analysis

Analytical data from this study was obtained from at least 3 biological replicates. Statistical analysis was performed by either one-way or two-way analysis of variance (ANOVA; RRID:SCR_002427) methods while the *post hoc* Tukey test was performed using the SPSS program (IBM, United States; RRID:SCR_002865). The results of these analyses are presented as letters on the relevant histograms. The same letter indicates a non-statistically significant difference (*p* > 0.05), whereas a different letter indicates a statistically significant difference (*p* < 0.05).

## Results

### Callus Induction From Mature *Setaria viridis* Seeds

**FIGURE 1:**
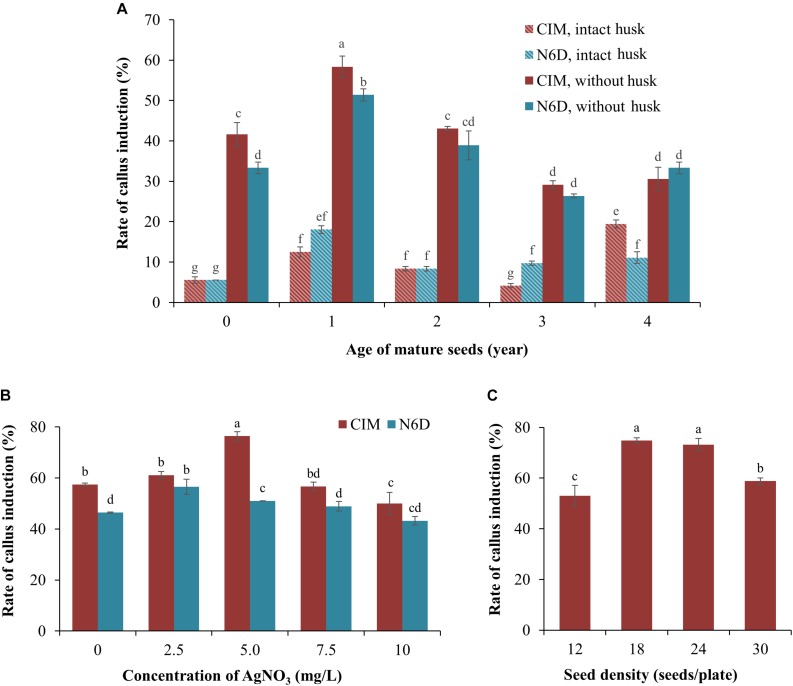
Improvement of callus induction from mature *Setaria viridis* seeds. **(A)** The effect of media and seed age on the rate of callus induction. Two medium compositions (CIM and N6D media) were used to test the rate of callus induction from intact and de-husked mature seeds stored at 4°C for different periods of time, ranging from freshly harvested seeds (0 year-old) to seeds that had been stored at 4°C for 4 years. **(B)** Effect of silver nitrate on the prevention of callus necrosis. CIM and N6D media supplemented with 0.0 to 10 mg/L AgNO_3_ was assessed to determine the impact of AgNO_3_ addition on callus quality. **(C)** The effect of different seed densities on the rate of callus induction was also assessed. The results of statistical analysis are presented as letters on the histograms. The same letter indicates a non-statistically significant difference (*p* > 0.05), whereas a different letter indicates a statistically significant difference (*p* < 0.05).

One of the most critical factors for the successful establishment of a robust *Agrobacterium*-mediated transformation protocol of a target species is the ability to identify a cell or tissue type from which high quality callus can be generated (Wang et al., 2011). Initially, freshly harvested (0 years of storage at 4°C), and 1-, 2-, 3-, and 4-year-old seeds that had been harvested from mature *S. viridis* wild-type plants, with or without their seed husk (consisting of lemma and palea), were cultured on either CIM or N6D medium plates to assess these multiple factors in parallel to determine which combination returned the highest efficiency of callus induction. After 1 week of culturing, callus initiation and proliferation were observed on both assessed types of culture media (Figure 1A). However, Figure 1A also shows that callus induction from seeds with an intact husk was relatively low, less than 20%, irrespective of the culture media used. In direct contrast, removal of the seed husks greatly increased the rate of callus induction on both types of culture media. The age (the period of time in years that wild-type *S. viridis* seeds were stored at 4°C) of the dehusked seeds was also demonstrated to strongly influence the rate of callus induction, with the highest induction rate achieved when 1-year-old dehusked seeds were used as the starting material on both CIM (58.3%) and N6D (51.4%) media. Lower callus induction frequencies, ranging from 26.4 to 43.1%, were observed when freshly harvested, 2-, 3-, and 4-year-old dehusked seeds were cultured on either CIM or N6D medium (Figure 1A). Furthermore, except for 4-year-old dehusked seeds, the efficiency of callus induction from seeds that had their husks removed, was repeatedly demonstrated to be higher on CIM than on N6D medium.

In an attempt to enhance both the quality and quantity of the callus material obtained from 1-year-old dehusked seeds, silver nitrate (AgNO_3_) was added to the culture medium (Figure 1B). The addition of 2.5 mg/L AgNO_3_ to the N6D medium returned the highest rate of callus induction, at 56.4%, whereas the addition of 7.5 and 10.0 mg/L AgNO_3_ to the N6D medium failed to improve callus induction from 1-year-old dehusked seeds. In addition, the quality of the callus material obtained from N6D medium supplemented with AgNO_3_ remained unchanged from those pieces of callus generated on non-supplemented N6D medium (data not shown). In direct contrast to this finding, the efficiency of callus induction on CIM medium was further enhanced via the addition of 5.0 mg/L of AgNO_3_, increasing callus induction from 57.1 to 76.4% (Figure 1B). However, the addition of higher concentrations of AgNO_3_, namely 7.5 and 10.0 mg/L, failed to promote greater callus induction percentages than that obtained for CIM medium supplemented with 5.0 mg/L AgNO_3_. Furthermore, calli generated on CIM medium supplemented with 5.0 mg/L AgNO_3_ also showed a healthy physical appearance (creamy yellow in colour, non-friable, hard and compact undifferentiated cells), and maintained their viability and proliferation properties in the subsequent steps of tissue culture.

Post the determination that the highest efficiency of callus induction was achieved with culturing dehusked, 1-year-old seeds on CIM medium supplemented with 5.0 mg/L AgNO_3_, the influence of seed density on callus induction was next assessed. Specifically, 12, 18, 24, and 30 dehusked, 1-year old seeds were transferred to culture plates containing CIM medium supplemented with 5.0 mg/L AgNO_3_. Interestingly, culture plates with only 12 seeds returned the lowest rate of callus induction at 53.0% (Figure 1C). Plates with a seed density of either 18 or 24 seeds per plate returned the highest levels of callus induction, 74.8 and 73.2%, respectively (Figure 1C). However, a seed density of 18 seeds per culture plate was selected for use in all subsequent experimentation with the callus material generated from culture plates with 18 seeds per plate expressing two additional and desirable phenotypes. Specifically, the calli generated from culture plates with 18 seeds per plate were considerably larger in physical size and composed of a highly compacted cellular mass (data not shown). These characteristics were not observed in the callus that was generated from culture plates that contained 24 seeds per plates, even though the frequency of callus induction was comparable between the culture plates with seed densities of 18 and 24 seeds per culture plate.

Together, the data presented in Figure 1 determined a series of parameters that collectively allowed for the efficient induction and subsequent generation of high quality callus material from mature *S. viridis* seeds, including; (1) the use of 1-year old seeds with husks removed; (2) plating out a seed density of 18 seeds per culture plate, and; (3) culturing 1-year-old dehusked *S. viridis* seeds on CIM medium supplemented with 5.0 mg/L AgNO_3_.

### *Agrobacterium* Co-cultivation With *Setaria viridis* Callus and Plantlet Regeneration

Post the production of healthy callus material, the method of callus co-cultivation with an *Agrobacterium* suspension that harboured the pANIC12A plant expression vector was assessed for further optimisation of the transformation protocol. Three distinct co-cultivation approaches were compared, namely the ‘dry,’ ‘semi-dry,’ and ‘wet’ co-cultivation approaches.

**FIGURE 2:**
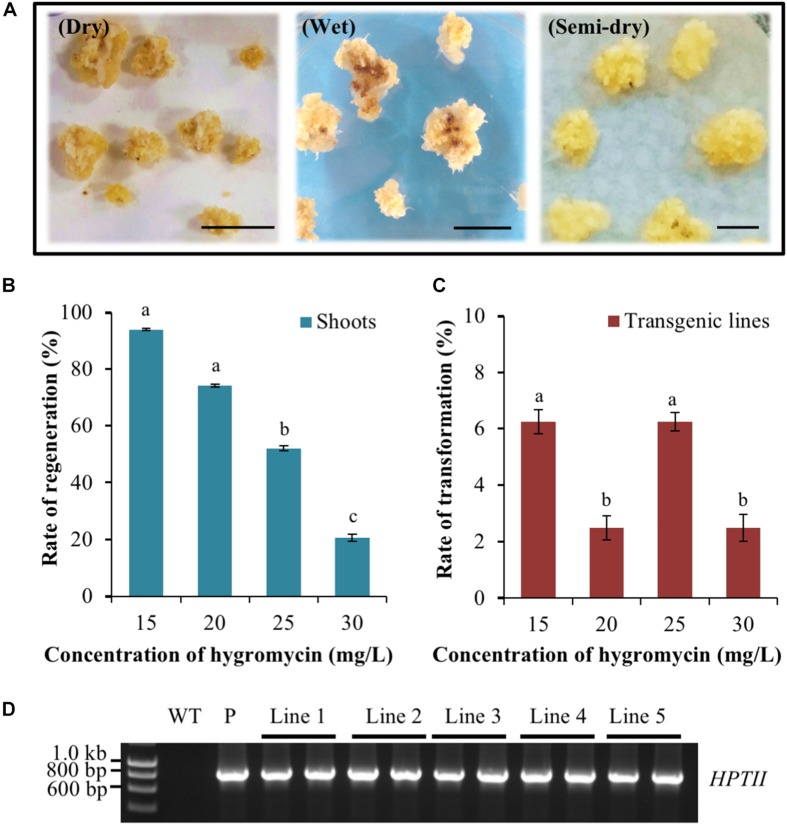
Assessment of the *Agrobacterium* co-cultivation approach on *Setaria viridis* transformation efficiency and the use of hygromycin for *in planta* transformation selection. **(A)** Dry co-cultivation involved incubation of transfected calli on sterile filter paper discs. Wet co-cultivation involved incubation of calli with *Agrobacterium* on CIMC medium and additional washing steps to remove excess *Agrobacterium*. Semi-dry co-cultivation involved incubation of transfected calli on CIMC medium overlaid with filter paper discs. *S. viridis* calli showed the highest rate of recovery and optimal growth characteristics using the semi-dry co-cultivation approach. Bar = 0.5 cm. **(B,C)** The rate of shoot regeneration was the highest at 15 mg/L hygromycin, 92%, but this rate dropped substantially to 20% when the growth medium was supplemented with 30 mg/L hygromycin. Media supplemented with 25 mg/L hygromycin showed the most efficient reduction of non-transformant events while additionally allowing for the obtainment of the highest rates of genuine transformation events, at 6.3%. The results of statistical analysis are presented as letters on the histograms. The same letter indicates a non-statistically significant difference (*p* > 0.05), whereas a different letter indicates a statistically significant difference (*p* < 0.05). **(D)** PCR amplification analysis confirmed the presence of the pANIC12A selectable marker gene, *HYGROMYCIN PHOSPHOTRANSFERASE II* (*HPTII*; a 845 bp amplicon) in each assessed *S. viridis* putative transformant (transformant lines 1 to 5). WT: the non-transformed *S. viridis* wild-type plant (negative PCR control). P: pANIC12A plasmid DNA (positive PCR control).

In the dry co-cultivation approach, the callus material was allowed to air-dry via its placement on sterilised filter paper for 3 days after incubation with the *Agrobacterium* suspension. However, the vast majority of callus material resulting from the dry co-cultivation approach displayed an overall reduction in size and also a change in colouration (dark yellowing and/or browning) post the 3-day ‘drying’ period on sterilised filter paper (Figure 2A). In addition, many of these calli also went on to subsequently develop necrosis, thus greatly reducing the frequency of recovering putatively transformed material. A low rate of recovery was also obtained using the wet co-cultivation approach. The long period of callus exposure to the *Agrobacterium* suspension resulted in high levels of *Agrobacterium* overgrowth. This in turn, required many additional washing steps to successfully remove the excess *Agrobacterium*, a process that damaged the callus. Damage to the callus material was evidenced by the high degree of soft, watery and/or necrotic regions that subsequently formed on the callus (Figure 2A) with similar observations previously reported for rice callus when using the same approach (Priya et al., 2012). In contrast to the dry and wet co-cultivation strategies, the callus material resulting from semi-dry co-cultivation continued to proliferate normally. Furthermore, this callus population maintained a healthy appearance when transferred to the selective medium. More specifically, the calli remained intact, were hard and dense, and retained a creamy yellow colour (Figure 2A).

Having established that the semi-dry co-cultivation approach readily allowed for the generation of healthy calli, the optimal concentration of the selective agent, hygromycin, that would enable the identification of only ‘true’ transformation events was investigated. Following 10–15 days of cultivation on plant regeneration medium, green foci that subsequently developed into shoot material were observed on calli cultivated on medium containing various concentrations of hygromycin. Subsequent molecular analyses (PCR and Southern blotting) identified two hygromycin concentrations, 15 and 25 mg/L, that returned the highest rate of genuine transformation events, at 6.3% (Figures 2B–D). Furthermore, and although both the 15 and 25 mg/L concentrations of hygromycin were determined to return a similar frequency of genuine transformation events, 25 mg/L of hygromycin was much more effective at eliminating false positives, that is; regenerated plant material (shoot and/or root material) that was subsequently determined to not harbour the introduced T-DNA segment of the pANIC12A plant expression vector. More specifically, 92.0% of calli produced shoot material on regeneration medium that contained 15 mg/L hygromycin, whereas this number decreased considerably to 54.1% for calli that were cultivated on regeneration medium that contained 25 mg/L hygromycin.

### Rapid Identification of Transformed Plant Material via the Use of a Visual Reporter

**FIGURE 3:**
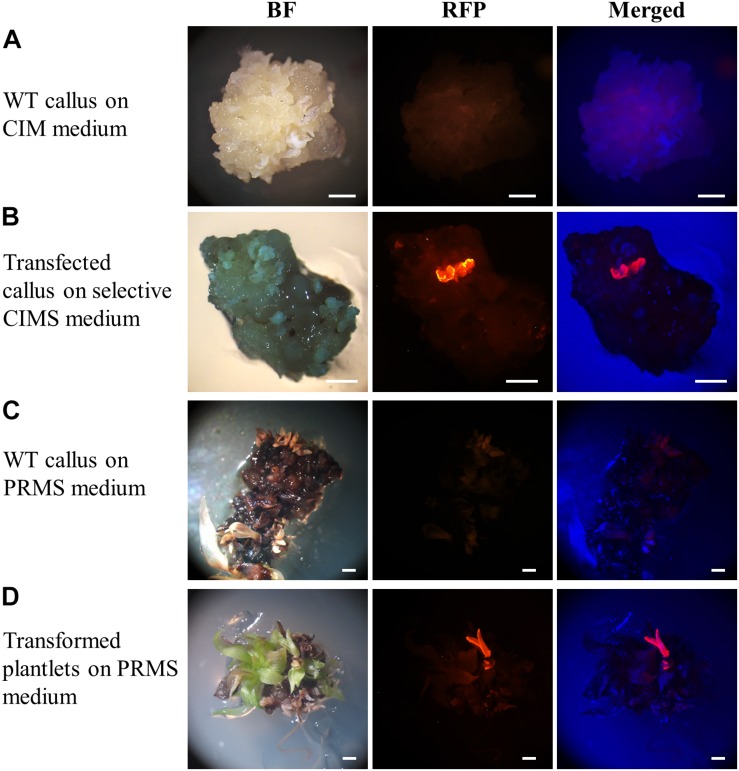
Representative dissecting microscopy images of wild-type *S. viridis* and putative *S. viridis* transformation lines. Wild-type *S. viridis* callus cultured on CIM medium showed no RFP fluorescence while a red fluorescent signal was readily observable for the hygromycin resistant callus material. Similarly, RFP fluorescence was only visually detectable in plantlets putatively transformed with the pANIC12A plant expression vector. Bar = 1 mm.

At both the callus and plantlet stage of the transformation process, the expression of the *RED FLUORESCENT PROTEIN* (*RFP*) reporter gene harboured by the pANIC12A plant expression vector was visually screened via fluorescence microscopy for rapid identification of putative transformant lines. When viewed under fluorescence microscopy, callus that did not harbour the introduced pANIC12A plant expression vector displayed a dull orange colouration, a colour indicative of auto-fluorescence (Figure 3A). In direct contrast, segments of callus into which the T-DNA region of the pANIC12A vector had presumably successfully integrated, fluoresced a bright red colour that was readily distinguishable from the background auto-fluorescence (Figure 3B). Similar observations were made at a later stage in the transformation process, that is; (1) plant-like structures such as stems or leaves that originated from regions of callus that did not harbour the introduced transgene exhibited a weak orange fluorescence signal (Figure 3C), whereas; (2) shoot or root structures originating from callus regions presumed to have integrated the pANIC12A T-DNA, fluoresced bright red (Figure 3D).

**FIGURE 4:**
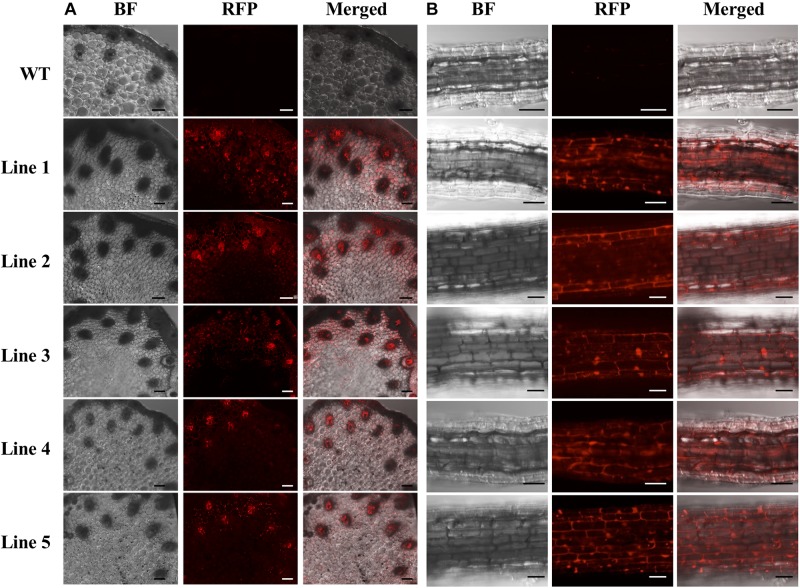
Cross sectional images of internode and root samples of T_1_ *S. viridis* transformant lines via confocal microscopy. Images were obtained using bright field (BF) microscopy and red fluorescence filters (RFP; 580 nm excitation, 630 nm emission) of internode **(A)** and root tissue **(B)** sections. Strong red fluorescence was observed in both the internodes and roots of transformant lines 1 to 5, however, no RFP signals were detected in non-transformed wild-type (WT) *S. viridis* samples. Bar = 100 μm.

Only the regenerated *S. viridis* plantlets that expressed RFP when visually assessed via fluorescence microscopy were continued through the remaining steps of the regeneration process. The putative T_0_ transformants, as determined via fluorescence microscopy, were allowed to progress through their entire developmental cycle (under standard glasshouse conditions) to allow for the generation and collection of T_1_ seeds. The T_1_ generation of plants was again visually assessed via fluorescence microscopy with RFP expression readily observable in the stem (Figure 4A) and root (Figure 4B) tissues of this transformant population. More specifically, in cross sections of the internodes of T_1_ plants, RFP was most highly expressed in the vascular bundles (Figure 4A). In roots sampled from T_1_ plants, red fluorescence was readily observed in the walls of most of the cells contained within each section, however, RFP expression did appear to concentrate at the periphery of the cells of the epidermal rind. In contrast, no RFP signal was detected in any sections sampled from wild-type plants when visualised under the same fluorescence microscopy settings (Figure 4; top panels).

### Molecular Analysis of Putative *Setaria viridis* Transformant Lines

**FIGURE 5:**
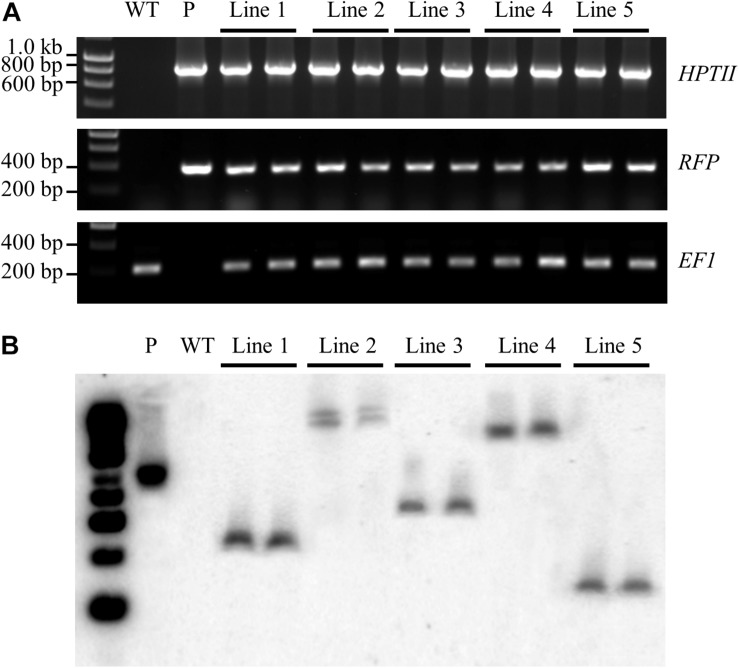
Confirmation of the stable integration of the pANIC12A transgene in *S. viridis* transformants via Southern blot hybridisation analysis. **(A)** PCR analyses confirmed the integration of two pANIC12A transgene-derived sequences in 5 T_1_ transformant lines, including the *HYGROMYCIN PHOSPHOTRANSFERASE II* [*HPTII* at 845 base pair (bp)] selectable marker gene and the *RED FLUORESCENT PROTEIN* (*RFP* at 360 bp) visual reporter gene. In addition, the *S. viridis* gene, *ELONGATION FACTOR 1* (*EF1* at 192 bp), was used as an internal control for PCR screening of *S. viridis* plant lines. **(B)** Southern blot hybridisation analysis revealed that transformant lines 1, 3, 4, and 5 harboured a single copy of the *HPTII* gene, as indicated by a single distinct band. However, two distinct bands were visualised for *S. viridis* transformant line 2 to indicate that this transformant line harboured multiple pANIC12A integration events. WT: the non-transformed *S. viridis* wild-type plant (negative PCR control). P: pANIC12A plasmid DNA (positive PCR control). Lines 1 to 5: *S. viridis* transformant line 1 to 5.

Prior to performing Southern blot hybridisation analysis, a standard PCR-based approach was applied to confirm that genomic DNA had been successfully extracted from all T_1_ transformant lines. An amplicon of the expected size [a 192-base pair (bp) product] was amplified for the internal control gene, *ELONGATION FACTOR 1* (*EF1*; *Sevir.J017000*) from non-transformed *S. viridis* wild-type plants and from each of the putative T_1_ transformant lines (Figure 5A). A PCR-based approach was further applied to screen for two transgene-specific sequences, namely the *HPTII* (a 845 bp amplicon) and *RFP* (a 360 bp amplicon) encoding genes. Figure 5A shows that both transgene targeted sequences were successfully amplified by PCR in all assessed T_1_ transformants, and as expected, failed to amplify from the wild-type sample. Together, PCR screening revealed that; (1) genomic DNA had been successfully extracted from all plant lines under analysis, and; (2) the T-DNA region of the pANIC12A plant expression vector had successfully integrated into the genome of each putative transformant line.

The isolated genomic DNA was next assessed via Southern blot hybridisation analysis to further confirm the successful integration of the pANIC12A derived T-DNA into the nuclear genomes of putative transformant lines, and to additionally determine the number of integration events that each putative transformant line harboured. Southern blot hybridisation analysis of two representative plants per T_1_ transformant line under assessment revealed a single hybridisation product for T_1_ transformant lines, 1, 3, 4, and 5 (Figure 5B). Southern blotting further confirmed that transformant line 2 harboured two integration events, with two distinct hybridisation products readily observable in both the T_1_ transformants (Figure 5B). Further, all hybridisation products returned for the 5 assessed transformant lines were of a distinct size. This finding strongly suggested that each plant line was representative of an independent integration event, that is; the T-DNA region of the pANIC12A plant expression vector had integrated into a unique chromosomal position in the *S. viridis* nuclear genome. In addition, expected hybridisation patterns were obtained for the *Pst*I-digested pANIC12A vector and wild-type genomic DNA samples, more specifically; a single hybridisation product of the expected size was observed for the vector control and no hybridisation signal was detected for the non-transformed *S. viridis* wild-type sample.

## Discussion

*Setaria viridis* has emerged as an ideal experimental model for the molecular genetic characterisation of C_4_ monocot grasses due to its small physical size, rapid life cycle, prolific seed production, simple growth requirements, and the public availability of an annotated genome sequence (Li and Brutnell, 2011; Bennetzen et al., 2012; Sebastian et al., 2014) and comprehensive transcriptomes (Martin et al., 2016; Studer et al., 2016). An essential component of the use of a specific species as an experimental model is the development of an efficient and reproducible protocol for the molecular modification of the selected species. *Agrobacterium*-mediated transformation using mature seed-derived callus has been routinely achieved in various other monocot species (Carvalho et al., 1997; Pola et al., 2009; Saika and Toki, 2010; Krishnan et al., 2013; Lin et al., 2017). These studies have repeatedly demonstrated that the generation of high quality and regenerable callus, the starting material for transformation, is the most crucial step for the development of an efficient *Agrobacterium*-mediated transformation protocol (Pola et al., 2009; Sahoo et al., 2011; Krishnan et al., 2013; Sah et al., 2014). Here we demonstrate that for *S. viridis*, the storage time and seed husk condition of mature seeds are critical factors for the generation of quality callus material, and in turn, a higher transformation efficiency. Culturing of mature seeds of differing age under identical conditions clearly revealed that the period of seed storage influenced the rate of callus induction. Furthermore, removal of the seed husk prior to the culturing of seeds of any age was demonstrated to dramatically improve the rate of callus induction (Figure 1A). Removal of the seed husk is thought to aid the seed in overcoming husk enhanced dormancy as well as presumably enhancing the rate of absorption of both nutrients and hormones from the culturing media (Bewley, 1997; Sebastian et al., 2014). As a result, mature seeds of *S. viridis* that had been stored at 4°C for a 1-year period, and dehusked prior to culturing on callus induction medium returned the highest rate of callus induction, approximately 57% (Figure 1A). Our result was consistent with previous studies on other members of the Poaceae family; that is, the efficiency of callus initiation was up to 83% when dehusked mature seeds were used as the starting material for callus induction (Sah et al., 2014; Lin et al., 2017).

Culture medium composition was also demonstrated to influence the callus induction rate from mature seeds. Specifically, the CIM medium was repeatedly shown to return higher rates of callus induction than the N6D medium. The efficiency of callus induction on the CIM medium was further improved from 57.4 to 76.4% via the addition of 5.0 mg/L AgNO_3_ (Figure 1B). This improvement was presumed to result from the inhibition of the formation and accumulation of ethylene and other phenolic compounds in the closed environment of tissue culturing, which in turn, promotes the formation of necrotic cells and tissues on the explant material being cultured (Kumar et al., 2009; Ahmad et al., 2013; Shah et al., 2014). The positive effect of the addition of AgNO_3_ to the culture media has previously been reported to improve the quality of the callus material derived from the culturing of pearl millet, sorghum (Oldach et al., 2001; Pola et al., 2009) and maize (Carvalho et al., 1997; Morshed et al., 2016). It is also important that the callus induction process is maintained in total darkness to obtain a healthy, larger and more compact mass of undifferentiated cells. Callus induced under illumination appeared to have a higher proportion of watery, sticky and necrotic cells, which is considered unsuitable material for efficient *Agrobacterium*-mediated transformation (Kumar et al., 2005; Pola et al., 2009).

Following callus induction, healthy calli were co-cultivated with *Agrobacterium* harbouring the pANIC12A plant expression vector. Amongst the three co-cultivation approaches assessed, the semi-dry method was determined to be the most suitable approach for the generation of callus material from *S. viridis* mature seed embryos. This method of co-cultivation circumvented the problem of nutrient deficiency and the accumulation of antimicrobial compounds at the sites of *Agrobacterium* infection caused by a hypersensitivity defence response associated with the dry method of co-cultivation (Hammond-Kosack and Jones, 1996; Kuta and Tripathi, 2005). In addition, the semi-dry co-cultivation approach also avoided the unwanted hypertonic conditions that result from high levels of *Agrobacterium* overgrowth and the additional washing steps associated with the wet co-cultivation approach (Hammond-Kosack and Jones, 1996; Kuta and Tripathi, 2005).

Establishment of the optimum concentration of the selective agent is another critical parameter that requires determination to ensure efficient and reproducible *Agrobacterium*-mediated transformation of a selected species. The pANIC12A plant expression vector used in this study contains a hygromycin (*HPTII*) resistance gene, and previous research has reported that *HPTII* is an effective selectable marker for *S. viridis* transformation (Martins et al., 2015a; Van Eck, 2018). Here, we show that all assessed hygromycin concentrations (15, 20, 25, and 30 mg/L) reduced the rate of recovery of non-transformed material (callus through to plantlets) during culturing. The percentage of *S. viridis* callus, which regenerated and subsequently developed into plantlets, rapidly dropped from 94 to 20%, via increasing the concentration of hygromycin in the selection media from 15 to 30 mg/L. This was believed to directly result from reduced numbers of somatic embryos that survived under the higher degree of selective pressure imposed (Zuraida et al., 2013). The toxicity of hygromycin was readily visible with the non-transformed callus material becoming shrunken in size and changing colour from yellow to either a brown or white colouration, or adopting an overall opaque appearance. In direct contrast, the putatively transformed callus material maintained their yellow colouration, and continued to actively divide on selective medium. After 2–3 weeks of cultivation on the regeneration medium, shoot and leaf material started to develop on most of the cultured callus, however, only genuinely transformed plantlets continued to grow and maintain a healthy appearance. Specifically, leaves and shoots retained a dark green colouration, and the root system vigorously developed and elongated after the transformed plantlets were transferred to the fresh selective regeneration medium. In direct contrast, the growth and development of the non-transformed plantlets (false positive individuals) was quickly inhibited, demonstrated by stunted growth of the regenerated plantlets and the development of necrotic tissues and/or bleached regions on shoots and leaves, at the higher concentrations of hygromycin (25 and 30 mg/L) used (Figures 2B,C). The hygromycin concentration, 25 mg/L, was determined to be the optimal concentration of this selective agent for *S*. *viridis* plants harbouring the pANIC12A-derived transgene, since 25 mg/L hygromycin concentration; (1) returned the highest rate of genuine transformation events, at 6.3%, and; (2) was the most effective at eliminating non-transformed, false-positives from the screening population at 54.1%. The results of this study were consistent with research reported by Van Eck et al. (2017), in which the transfected calli were regenerated on plant regeneration medium supplemented with 20 mg/L hygromycin, and that returned an average transformation efficiency of approximately 10%.

To further assist the screening of putative transformants, the pANIC12A plant expression vector was used for all transformation experiments reported here due to this vector harbouring the *RFP* gene from *Porites porites* whose expression is under the control of the switchgrass *UBIQUITIN1* (*UBI1*) promoter (Mann et al., 2012a). RFP fluorescence has been successfully utilised previously as a visual marker across a range of plant species, including switchgrass, rice, tomato, and *Arabidopsis* (Liu et al., 2011; Mann et al., 2012a, b). The inclusion of a fluorescent reporter within the plant expression vector offers a non-destructive screening approach for the rapid identification of putative transformant lines. RFP fluorescence was clearly visible at the callus, plantlet (Figure 3) and mature plant (Figure 4) stages of the transformation process in each of the plant lines subsequently demonstrated to be a genuine transformant via PCR-based transgene screening. In addition, Southern blot hybridisation analysis provided the most conclusive evidence to demonstrate the successful integration and stable inheritance of the T-DNA transgene from the pANIC12A plant expression vector into the *S. viridis* genome. Hybridisation results readily confirmed the stable integration of the pANIC12A T-DNA into the nuclear genome of each assessed T_1_ transformant line. A single T-DNA integration event was evident by visualisation of a single hybridisation product for 4 out of the 5 transformant lines analysed (Lines 1, 3, 4, and 5; Figure 5B). Furthermore, the visualisation of uniquely sized hybridisation products strongly suggested that each product was the result of an independent integration event.

## Conclusion

The difficulties associated with the successful induction of callus, and the subsequent regeneration of plant material from transformed callus material that stably harboured the introduced transgene, were considered a major hurdle in using *S. viridis* as an experimental model system for the continued molecular genetic characterisation of C_4_ monocot grasses. Here, an efficient, robust, and reproducible *Agrobacterium*-mediated transformation system was established for *S. viridis* A10. Together, the optimisation steps reported here greatly enhanced the frequency of callus induction, 76.4%, and the overall transformation efficiency rate, 6.3%. Further, the total timeframe of the established transformation system (from callus induction to the production of transformed plant lines) was relatively short at approximately 4 months. This study confirms the continued use of *S. viridis* as an attractive experimental model system for the molecular genetic characterisation of the C_4_ monocot grasses, and of particular importance, the findings made in *S. viridis* have the potential to be readily transferred to other agronomically important C_4_ crop species in future breeding programs.

## Data Availability Statement

All datasets generated for this study are included in the article/Supplementary Material.

## Author Contributions

CG proposed the study. DN performed *Agrobacterium*-mediated transformation, collected data, produced the Figures, and drafted the manuscript. DN, AE, and CG analysed data and improved the protocol. JV provided further advice for protocol improvement. AE, JV, and CG revised the manuscript.

## Conflict of Interest

The authors declare that the research was conducted in the absence of any commercial or financial relationships that could be construed as a potential conflict of interest.

## Supplementary Material

APPENDIX S1 Table of *Setaria viridis* transformation media.

APPENDIX S2 Schematic representation of the pANIC12A vector.

APPENDIX S3 Table of stock buffers.
