# Supplementary material for: Robust and Reproducible Agrobacterium-Mediated Transformation System of the C4 Genetic Model Species Setaria viridis
Source: Front Plant Sci. 2020 Mar 13;11:281. doi: 10.3389/fpls.2020.00281 (PMC7082778; doi:10.3389/fpls.2020.00281)
Supplement: APPENDIX S1 — Table of Setaria viridis transformation media. [file Data_Sheet_1.docx]

Appendix 1

Robust and reproducible *Agrobacterium*-mediated transformation system of the C_4_ genetic model species *Setaria viridis*

Duc Quan Nguyen^1^, Joyce Van Eck^2,3^, Andrew L. Eamens^1†^ and Christopher P. L. Grof^1*†^

^1^ Centre for Plant Science, School of Environmental and Life Sciences, University of Newcastle, Callaghan NSW 2308, Australia

^2^ Boyce Thompson Institute, Ithaca, NY, United States

^3^ Plant Breeding and Genetics Section, School of Integrative Plant Science, Cornell University, Ithaca, NY, United States

*** Correspondence:**
Christopher Grof
[chris.grof@newcastle.edu.au](mailto:chris.grof@newcastle.edu.au)

**†** These authors contributed equally to this work

**Table A1: *Setaria viridis* transformation media used at various stages.** Media were sterilised by autoclaving at 121^o^C for 20 minutes, and stored at 4^o^C.

| **Media** | **Ingredients (per Litre)** |
| --- | --- |
| **YEP** | 10 g peptone, 10 g yeast extract, 5.0 g NaCl. After autoclaving, 100 mg ampicillin, 50 mg kanamycin and 25 mg rifampicin were added to the medium. Medium was dispensed into 100 x 10 mm petri dishes. |
| **Callus induction medium (CIM)** | 4.3 g MS salts, 5.0 ml MS vitamins stock, 40 g maltose, 35 mg ZnSO_4_.7H_2_O, 0.6 mg CuSO_4_.5H_2_O, 2.0 mg 2,4-D, 0.5 mg kinetin and 4.0 g Gelzan^TM^. pH 5.8. Medium was dispensed into 100 x 20 mm petri dishes. |
| **200X MS Vitamins stock** | 20 g/L myo-inositol, 100 mg/L nicotinic acid, 100 mg/L pyridoxine HCl, 20 mg/L thiamine HCl and 400 mg/L glycine. Filter sterilisation. |
| **CIMA medium** | CIM medium supplemented with 5.0 mg/L of AgNO_3_. Medium was dispensed into 100 x 20 mm petri dishes. |
| **N6D medium** | 20 mL **Stock 1** (23.16 g/L (NH_4_)SO_4_, 20 g/L KH_2_PO_4_, 0.166 g/L MnSO_4_.4H_2_O, 0.074 g/L ZnSO_4_.7H_2_O, 0.04 g/L KI, 0.08 g/L H_3_BO_4_, 0.5 mg/L CoCl.6H_2_O, 0.05 mg/L CuSO_4_.5H_2_O and 0.05 mg/L Na_2_MoO_4_.2H_2_O), 5.0 mL **Stock 2** (33.2 g/L CaCl_2_.2H_2_O), 5 mL **Stock 3** (37 g/L MgSO_4_.7H_2_O), 5.0 mL **Stock 4** (0.112 g/L FeSO_4_.7H_2_O and 0.1492 g/L Na_2_.EDTA) , 5.0 mL **Stock 5** (0.04 g/L nicotinic acid, 0.4 g/L thiamine HCl, 0.04 g pyridoxine HCl and 4.0 g/L myo-inositol), 2.83 g KNO_3_, 30 g sucrose, 2.88 g L-proline, 300 mg casein hydrolysate, 4.0 g phytagen, 2.0 mg 2,4-D. pH 5.8. Medium was dispensed into 100 x 20 mm petri dishes. |
| **Co-cultivation medium (CIMC)** | 4.3 g MS salts, 5.0 ml 200X MS vitamins stock, 40 g maltose, 35 mg ZnSO_4_.7H_2_O, 0.6 mg CuSO_4_.5H_2_O, 4.0 g Gelzan^TM^. pH 5.8. After autoclaving, 1.0 mL 200 μM acetosyringone was added to the medium. Medium was dispensed into 100 x 20 mm petri dishes. |
| **CIM selective medium (CIMS)** | CIM medium supplemented with 40 mg/L hygromycin and 150 mg Timentin. Antibiotics were added to the medium after autoclaving. Medium was dispensed into 100 x 20 mm petri dishes. |
| **Plant regeneration medium (PRM)** | 4.3 g MS salts, 5.0 ml 200X MS vitamins, 20 g sucrose, 2 mg kinetin, and 7.0 g phytoblend. pH 5.8. Medium was dispensed into 100 x 20 mm petri dishes. |
| **PRM selective medium (PRMS)** | PRM medium supplemented with 25 mg/L hygromycin and 150 mg/L Timentin. Antibiotics were added to the medium after autoclaving. Medium was dispensed into 100 x 20 mm petri dishes. |
| **Root induction medium (RM)** | 2.15 g MS salts, 5.0 ml 200X MS vitamins, 30 g sucrose and 7.0 g phytoblend. pH 5.8. Medium was dispensed into Magenta^TM^ GA-7 vessels. |
| **RM selective medium (RMS)** | RM medium supplemented with 25 mg/L hygromycin and 150 mg/L Timentin. Antibiotics were added to the medium after autoclaving. Medium was dispensed into Magenta^TM^ GA-7 vessels. |
