# Supplementary material for: Robust and Reproducible Agrobacterium-Mediated Transformation System of the C4 Genetic Model Species Setaria viridis
Source: Front Plant Sci. 2020 Mar 13;11:281. doi: 10.3389/fpls.2020.00281 (PMC7082778; doi:10.3389/fpls.2020.00281)
Supplement: APPENDIX S2 — Schematic representation of the pANIC12A vector. [file Data_Sheet_2.docx]

Appendix 2

Robust and reproducible *Agrobacterium*-mediated transformation system of the C_4_ genetic model species *Setaria viridis*

Duc Quan Nguyen^1^, Joyce Van Eck^2,3^, Andrew L. Eamens^1†^ and Christopher P. L. Grof^1*†^

^1^ Centre for Plant Science, School of Environmental and Life Sciences, University of Newcastle, Callaghan NSW 2308, Australia

^2^ Boyce Thompson Institute, Ithaca, NY, United States

^3^ Plant Breeding and Genetics Section, School of Integrative Plant Science, Cornell University, Ithaca, NY, United States

*** Correspondence:**
Christopher Grof
[chris.grof@newcastle.edu.au](mailto:chris.grof@newcastle.edu.au)

**†** These authors contributed equally to this work


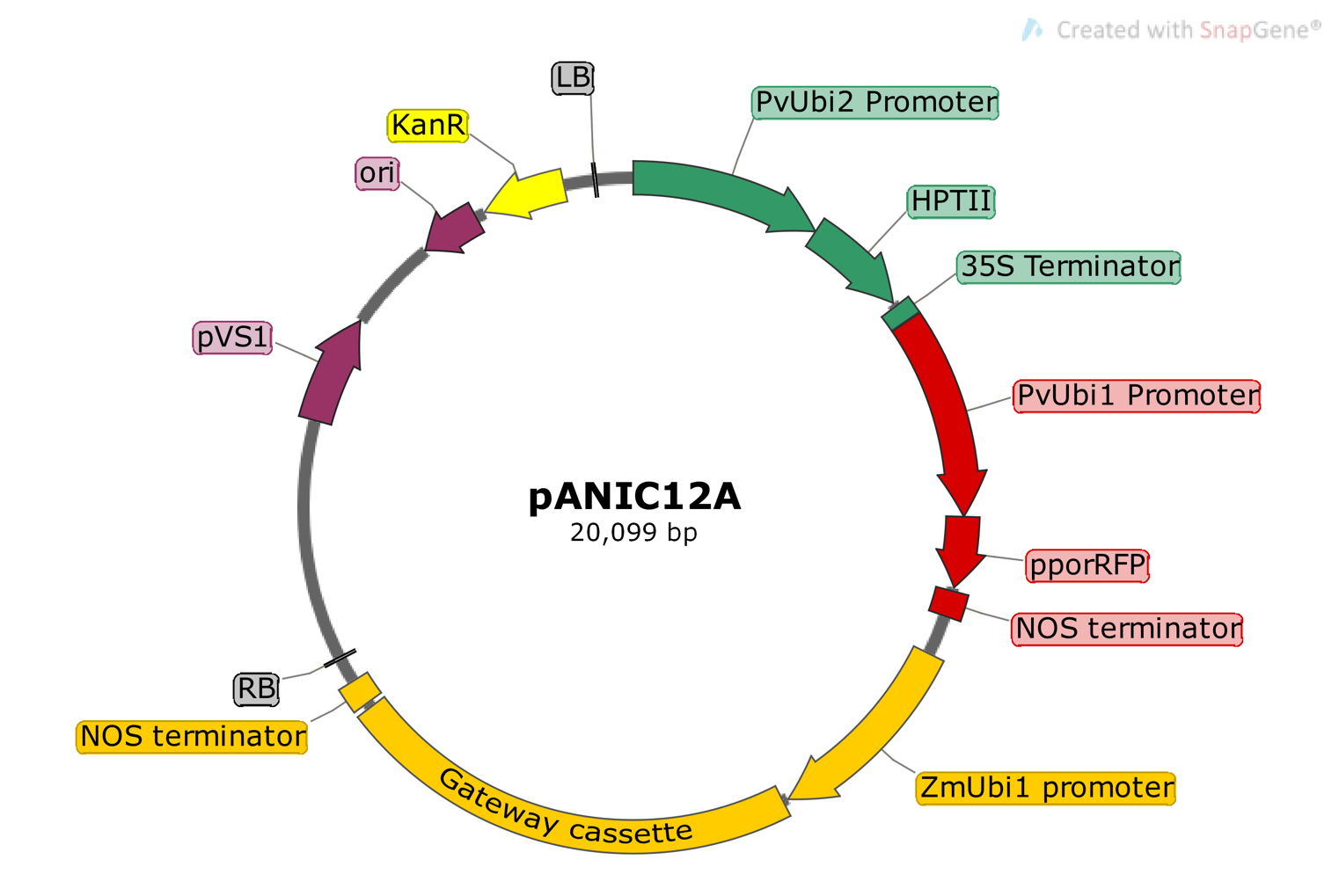


**Figure A1: Schematic representation of the pANIC12A vector.** *PvUBI1* and *PvUBI2*, *Panicum virgatum* ubiquitin 1 and 2 promoters; *ZmUbi1,* maize ubiquitin 1 promoter; *HPTII*, *HYGROMYCIN B PHOSPHOTRANSFERASE*; *pporRFP*, *Porites porites RED FLUORESCENT PROTEIN*; 35S, *Cauliflower mosaic virus* 35S terminator; NOS, *A. tumefaciens* *Nos* terminator; LB, left border; RB, right border; pVS1, *A. tumefaciens* origin of replication; Ori, *E. coli* origin of replication; *Kan^R^*, kanamycin bacterial resistance gene (Mann et al., 2012).

Mann, D.G.J, Lafayette, P.R., Abercrombie, L.L., King, Z.R., Mazarei, M., Halter, M.C., Poovaiah, C.R., Baxter, H., Shen, H., Dixon, R.A., Parrott, W.A., and Neal Stewart, C.Jr. (2012). Gateway-compatible vectors for high-throughput gene functional analysis in switchgrass (*Panicum virgatum* L.) and other monocot species. J. Plant Biotechnol. 10, 226-236.
