# Supplementary material for: Robust and Reproducible Agrobacterium-Mediated Transformation System of the C4 Genetic Model Species Setaria viridis
Source: Front Plant Sci. 2020 Mar 13;11:281. doi: 10.3389/fpls.2020.00281 (PMC7082778; doi:10.3389/fpls.2020.00281)
Supplement: APPENDIX S3 — Table of stock buffers. [file Data_Sheet_3.docx]

Appendix 3

Robust and reproducible *Agrobacterium*-mediated transformation system of the C_4_ genetic model species *Setaria viridis*

Duc Quan Nguyen^1^, Joyce Van Eck^2,3^, Andrew L. Eamens^1†^ and Christopher P. L. Grof^1*†^

^1^ Centre for Plant Science, School of Environmental and Life Sciences, University of Newcastle, Callaghan NSW 2308, Australia

^2^ Boyce Thompson Institute, Ithaca, NY, United States

^3^ Plant Breeding and Genetics Section, School of Integrative Plant Science, Cornell University, Ithaca, NY, United States

*** Correspondence:**
Christopher Grof
[chris.grof@newcastle.edu.au](mailto:chris.grof@newcastle.edu.au)

**†** These authors contributed equally to this work

**Table A2: Stock buffers used at various stages.** All buffers were stored at room temperature.

| **Stock buffers** | **Ingredients (per Litre)** |
| --- | --- |
| **1M Tris-HCl** | 121 g Tris base, 42 mL HCl, pH 8.0. |
| **0.5 M Phosphate buffer (PB)** | 234 mL NaH_2_PO_4_.2H_2_O stock solution (dissolve 69 g in 1 L of H_2_O), 766 mL of Na_2_HPO_4_.7H_2_O stock solution (dissolve 134 g in 1 L of H_2_O), pH 7.2. |
| **20X Saline sodium citrate (SSC)** | 175 g NaCl, 88.25 g Tri-sodium citrate, pH 7.0. |
| **1% TBE** | 220 mM Tris, 180 mM Borate, 5 mM EDTA, pH 8.3. |
| **Depurination solution** | 0.25 M HCl. |
| **Denaturation solution** | 0.5 M NaOH, 1.5 M NaCl. |
| **Neutralisation solution** | 0.5 M Tris-HCl, 1.5 M NaCl; pH 7.5. |
| **Church and Gilbert solution** | 0.5 M phosphate buffer (pH 7.2), 7% w/v SDS, 10 mM EDTA, 0.1 mg/mL BSA, 0.01 mg/mL salmon sperm DNA sodium salt (Sigma-Aldrich, Missouri, USA). |
| **Low stringency buffer** | 2X SSC, 0.1% w/v SDS. |
| **Medium stringency buffer** | 1X SSC, 0.1% w/v SDS. |
| **High stringency buffer** | 0.1X SSC, 0.1% w/v SDS. |
